# Potential side effects of antibacterial coatings in orthopaedic implants: A systematic review of clinical studies

**DOI:** 10.3389/fbioe.2023.1111386

**Published:** 2023-02-09

**Authors:** Hua Li, Daofeng Wang, Wupeng Zhang, Gaoxiang Xu, Cheng Xu, Wanheng Liu, Jiantao Li

**Affiliations:** ^1^ Senior Department of Orthopedics, The Fourth Medical Center of Chinese PLA General Hospital, Beijing, China; ^2^ National Clinical Research Center for Orthopedics, Sports Medicine and Rehabilitation, Beijing, China; ^3^ School of Medicine, Nankai University, Tianjin, China

**Keywords:** antibacterial coatings, clinical studies, side effect, implant, systematic review

## Abstract

**Objective:** The systematic review aimed to determine the potential side effects of antibacterial coatings in orthopaedic implants.

**Methods:** Publications were searched in the databases of Embase, PubMed, Web of Science and Cochrane Library using predetermined keywords up to 31 October 2022. Clinical studies reporting side effects of the surface or coating materials were included.

**Results:** A total of 23 studies (20 cohort studies and three case reports) reporting the concerns about the side effects of antibacterial coatings were identified. Three types of coating materials, silver, iodine and gentamicin were included. All of studies raised the concerns regarding safety of antibacterial coatings, and the occurrence of adverse events was observed in seven studies. The main side effect of silver coatings was the development of argyria. For iodine coatings, only one anaphylactic case was reported as an adverse event. No systemic or other general side effects were reported for gentamicin.

**Conclusion:** Clinical studies on the side effects of antibacterial coatings were limited. Based on the available outcomes, the most reported side effects of antibacterial coatings in clinical use were argyria with silver coatings. However, researchers should always pay attention to the potential side effects of antibacterial materials, such as systematic or local toxicity and allergy.

## 1 Introduction

Implant-related infection (IRI) is one of the most devastating complications after orthopaedic procedures (Van Belleghem et al., 2020). The frequency of IRI after arthroplasty ranges from 0.5% to 15% (Cats-Baril et al., 2013; Lenguerrand et al., 2017). Spinal implant infection affects between 2% and 13% of patients (McClelland et al., 2016). Postoperative infection following trauma surgeries has an incidence of 0.5%–50% (Bonnevialle et al., 2012; Oliveira et al., 2016). With some reporting an annual number of 1,000,000 IRIs occurring in the United States (US), over $1.6 billion is spent for the treatment against the IRI (Edmiston et al., 2011). The socio-economic burden of IRI is heavy, with relatively high morbidity and mortality (Berbari et al., 2012).

Previous studies have demonstrated that the formation of biofilm plays an important role in the pathogenesis of IRI (Josse et al., 2019; van Vugt et al., 2019). In general, there are two important steps in the formation of biofilm. Initially, the bacteria attach to the implant surface through physicochemical interactions. Subsequently, the bacteria replicate to form multilayered cell colonies on the surface through molecular and cellular interactions, producing an extracellular matrix forming a complex community called biofilm (Costerton et al., 1999). The formation of biofilm can render the bacteria extremely resistant to the human immune system and antibiotics (Zimmerli et al., 2004; Gbejuade et al., 2015; Riool et al., 2017). Gristina et al. coined the term “race for the surface” to illustrate the competition between host cells and bacteria for adhesion to the surface (Gristina et al., 1988). This concept leads to a promising strategy of modifying the implant surface with antibacterial coatings.

Numerous studies have examined the ability of antibacterial-coated implants against infections. Many of these studies have demonstrated excellent antibacterial properties (Shirai et al., 2016; Wilding et al., 2016; Hardes et al., 2017). Sambri et al. investigated the use of silver-coated megaprostheses *versus* uncoated megaprostheses in patients with tumor prostheses infections and found that the reinfection rate in coated group was lower than that in uncoated group (10.3% VS. 17.5%) (Sambri et al., 2020). Kabata et al. used an iodine-coated hip implant for 28 patients who had IRI, pyogenic arthritis or immunosuppressive condition. In their cohort, no signs of infection were observed after a 3-year follow up (Kabata et al., 2015). Savvidou et al. conducted a systematic review and meta-analysis on the efficacy of antibacterial surface in preventing IRI. The authors included seven comparative studies regarding different antibacterial coatings and found that implants with antibacterial coatings could reduce the risk of infection with an odds ratio of 2.9 as compared with general implants (Savvidou et al., 2020).

However, the coating procedure should ensure not only antibacterial resistance but also safety, as antibacterial materials may be toxic to host cells. In this regard, a few clinical studies focused on the potential side effects of the coated surface. To our knowledge, few systematic reviews have been published on this subject. Alt analyzed the risk and benefit of antibacterial coatings using the method of systematic review. Nevertheless, the author focused the gentamicin- and silver-coated implants only at the initial search and did not complete a comprehensive search. Studies reporting the potential side effects of antibacterial coatings may be missed, as many materials have been used to modify the implant surface (Alt, 2017). Therefore, the aim of this systematic review was to ascertain the potential side effects of antibacterial-coated implants reported in clinical studies.

## 2 Methods

This study was registered in the International Prospective Register of Systematic Reviews (PROSPERO) (CRD42018102464). This systematic review was conducted according to the Preferred Reporting Items for Systematic reviews and Meta-Analyses (PRISMA) Statement protocol (Shamseer et al., 2015).

### 2.1 Search strategy

Embase, PubMed, Web of Science and the Cochrane library databases were searched up to October 2022. The search keywords included antibacterial, coating and orthopaedic implants (Supplementary Table S1). We developed specific search strategies for each database. The bibliographies of included articles and relevant review articles were also assessed for potential eligibility.

### 2.2 Eligibility criteria and study selection

The inclusion criteria encompassed the following: 1) clinical studies or case reports regarding the antibacterial surface for orthopaedic surgeries; 2) modifying the implant surface using physical and/or chemical methods; 3) outcomes including the data of any side effect with regard to the surface or coating materials. We excluded studies that imparted antibacterial materials directly to the implant surface using cement or hydrogel which carried these materials, because these two materials were added to implant surface by surgeons freehand, which could jeopardize the uniformity and consistence. Non-English language publications, *in vitro* studies, brief reports, reviews and conference proceedings were also excluded. After dropping the duplicates, two authors reviewed the titles and abstracts to identify potentially eligible studies independently. Full texts were then read independently by the same two authors to determine the final list of included studies. A senior doctor was consulted for the final consensus if there occurred a disagreement.

### 2.3 Data extraction

The primary goal of this systematic review was to determine the side effects of antibacterial coatings against the IRI. Therefore, we extracted any possible negative results as a consequence of antibacterial modification. Other information was also extracted, including the year of publication, study design, type of surface coating, coating procedure, load of antibacterial materials, patients’ attributes and orthopaedic procedure. We contacted the corresponding author in an attempt to obtain any additional unclear or missing data.

### 2.4 Assessment of quality and bias

Two authors estimated the quality of the included studies independently. For cohort studies, the Newcastle-Ottawa Scale was used (Stang, 2010). For case reports, we applied the Joanna Briggs Institute Critical Appraisal Checklist (Munn et al., 2015; Wang et al., 2022), which has been widely used to assess the quality of case reports.

## 3 Results

### 3.1 Overview of study selection

**FIGURE 1:**
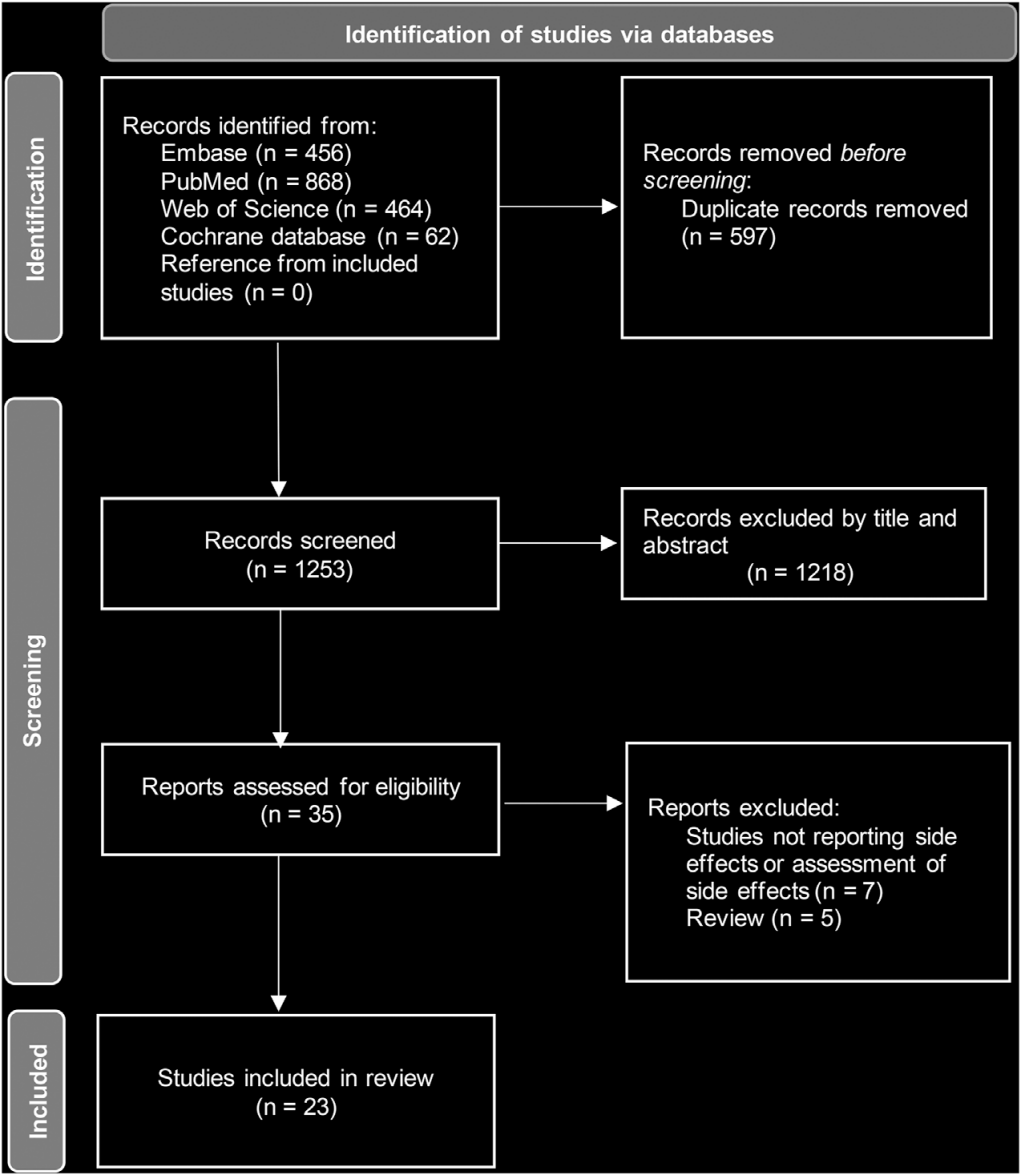
Flowchart of PRISMA.

Initially, a total of 1850 studies were identified. After removing duplicates, 1,253 studies were available for review. We screened the titles and abstracts and excluded 1,218 papers that did not meet the criteria. The full texts of the remaining 35 papers were read, and finally, 23 studies (20 cohort studies and three case reports) were included (Figure 1). Of these studies, 14 reported on silver coatings, six on iodine coatings and three on gentamicin coatings. These studies were mainly published from Japan (*n* = 8), followed by Germany (*n* = 6) and Italy (*n* = 5). All the studies regarding iodine coatings were from Japan. The coated implants were mainly used for tumor resections, open fractures, revision of prosthetic joint infection, arthroplasty of pyogenic arthritis and so on. The mean age of recruited patients ranged from 14 to 80 years (Table 1).

**TABLE 1:** Characteristics of the included studies.

Authors	Year	Country	Study design	Case number	Patient age (^a^)	Indications	Coating	Implant
Smolle et al	2022	Austria	Cohort study	46	47.1	ReTJA, Oncology	Silver	MUTARS megaprostheses
Denes et al	2022	France	Case report	1	75	Oncology	Silver	MUTARS megaprostheses
Hashimoto et al	2020	Japan	Case report	1	80	TJA of SA	Silver	Ag-HA TJA prostheses
Sambri et al	2020	Italy	Cohort study	68	30	PJI	Silver	PorAg megaprostheses
Shirai et al	2019	Japan	Cohort study	72	59.3	PJI, ReTJA, Spinal surgery, Fracture, Oncology	Iodine	Iodine-coated megaprostheses, spinal instruments, nails and TJA prostheses
Schmidmaier et al	2017	Germany	Cohort study	100	46.6	Fracture	Gentamicin	Tibial nails
Moghaddam et al	2016	Germany	Cohort study	25	50.9	Fracture	Gentamicin	Tibial nails
Eto et al	2016	Japan	Cohort study	20	77.0	TJA	Silver	Ag-HA TJA prostheses
Piccioli et al	2016	Italy	Cohort study	30	56.2	Oncology	Silver	MUTARS megaprostheses
Donati et al	2016	Italy	Cohort study	68	61.6	Oncology	Silver	MUTARS megaprostheses
Scoccianti et al	2016	Italy	Cohort study	33	55	ReTJA, Oncology	Silver	PorAg megaprostheses
Hayashi et al	2015	Japan	Cohort study	69	55.1	Spinal surgery	Iodine	Iodine-coated spinal instruments
Kabata et al	2015	Japan	Cohort study	30	56	PJI, TJA of SA	Iodine	Iodine-coated megaprostheses
Shirai et al	2014	Japan	Cohort study	47	53.6	Oncology, TJA of SA	Iodine	Iodine-coated megaprostheses
Shirai et al	2014	Japan	Cohort study	38	33.6	Fracture	Iodine	Iodine-coated pins
Karakasli et al	2014	Turkey	Case report	1	14	Oncology	Silver	MUTARS megaprostheses
Glehr et al	2013	Austria	Cohort study	32	46	ReTJA, Oncology	Silver	MUTARS megaprostheses
Hussmann et al	2013	Germany	Cohort study	18	60.1	ReTJA, Oncology	Silver	MUTARS megaprostheses
Tsuchiya et al	2012	Japan	Cohort study	222	49.4	PJI, TJA of SA	Iodine	Iodine-coated megaprostheses, spinal instruments, nails and TJA prostheses
Fuchs et al	2011	Germany	Cohort study	21	47.7	Fracture	Gentamicin	Tibial nails
Hardes et al	2010	Germany	Cohort study	51	37	Oncology	Silver	MUTARS megaprostheses
Hardes et al	2007	Germany	Cohort study	20	61.8	Oncology	Silver	MUTARS megaprostheses
Massè et al	2000	Italy	Cohort study	24	34.7	Fracture	silver	Silver-coated pins

^a^ , year; ReTJA, revision total joint arthroplasty; MUTARS, modular universal tumor and revision system; SA, septic arthritis; Ag-HA, silver oxide and hydroxyapatite; PJI, periprosthetic joint infection; PorAg, Porous Argentum.

### 3.2 Coating technologies

The included studies revealed four different antibacterial modification technologies with silver. Modular universal Tumor and Revision System (MUTARS) megaprostheses (Implantcast, Buxtehude, Germany) were modified by galvanic deposition of pure silver (Hardes et al., 2007; Hardes et al., 2010; Glehr et al., 2013; Hussmann et al., 2013; Karakasli et al., 2014; Donati et al., 2016; Piccioli et al., 2016; Denes et al., 2022; Smolle et al., 2022). A mean amount of 0.91 g (range 0.7–1.2 g) silver was coated on the implant surface. Porous Argentum (PorAg) MegaC prostheses (Waldemar, Hamburg, Germany) used the vapor deposition of TiAg_20_N to modify the surface with a silver content of 0.33 g (Scoccianti et al., 2016; Sambri et al., 2020). Another coating method, thermal plasma spraying, was used by Eto et al. to load a mixture of silver oxide and hydroxyapatite (Ag-HA) onto the implant surface, and only 0.003 g silver was added (Eto et al., 2016; Hashimoto et al., 2020). Massè et al. reported the clinical use of silver-coated stainless-steel pins for external fixation in patients with open fractures (Massè et al., 2000). Silver was coated to the pins based on ion-beam-assisted deposition from vapor.

Iodine coatings were produced by the Chiba Institute of Technology (Narashino, Japan) (Tsuchiya et al., 2012; Shirai et al., 2014a; Shirai et al., 2014b; Hayashi et al., 2015; Kabata et al., 2015; Shirai et al., 2019). This type of coating is an adhesive anodic oxide film, which forms through the anodization of povidone-iodine electrolyte. The thickness of the coating was between 5 and 10 μm with the capacity to support 10–12 μg/cm^2^ iodine.

Gentamicin-coated technology is now only used in tibia nails (Unreamed Tibial Nail [UTN] PROtect, DePuy Synthes, Bettlach, Switzerland; Expert Tibial Nail [ETN] PROtect, DePuy Synthes, Johnson & Johnson, New Brunswick, New Jersey) (Fuchs et al., 2011; Moghaddam et al., 2016; Schmidmaier et al., 2017). This coating consisted of a poly (L-lactic acid) (PLLA) matrix containing gentamicin sulphate. The surface modification was achieved by the dip coating process. The total amount of gentamicin on an implant ranged from 10 to 50 mg.

### 3.3 Concerns about side effects

All of the studies reported the concerns regarding the safety of antibacterial coatings and seven studies detected the development of side effects (Table 2).

**TABLE 2:** Reported side effects of antibacterial coatings.

Coating and concerns regarding safety	Implant	Encountered side effects
**Silver**: argyria, high blood silver levels, damage to organ function, suppression on osteointegration, neurotoxicity	MUTARS megaprostheses	Argyria: the incidence of 23% (7/32) in Glehr et al.‘s study, 8.7% (4/46) in Smolle et al.‘s study and 2.0% (1/51) in Hardes et al.‘s study; two cases in two case reports The other studies did not detect argyria and any other adverse events
	Ag-HA TJA prostheses	Not seen
	PorAg megaprostheses	Not seen
	Silver-coated pins	Increase in blood silver levels: PreOP, 0.20 μg/L; PostOP 3.12 (range 0.2–20.55) μg/L
**Iodine**: allergy, abnormality of thyroid function	Iodine-coated implants	Allergy: only one case among 222 patients in Tsuchiya et al.‘s study The other studies did not report any adverse events
**Gentamicin**: allergy, high gentamicin blood levels, nephrotoxicity, hepatotoxicity	Tibial nails	Not seen

MUTARS, modular universal tumor and revision system; Ag-HA, silver oxide and hydroxyapatite; TJA, total joint arthroplasty; PorAg, Porous Argentum. PreOP, preoperatively; PostOP, postoperatively.

#### 3.3.1 Silver coating

Fourteen studies reported concerns regarding the side effects of silver, including a high concentration of silver in blood, impairment of liver and/or kidney function, implication on ossification and osteointegration, argyria and neurotoxicity (Massè et al., 2000; Hardes et al., 2007; Hardes et al., 2010; Glehr et al., 2013; Hussmann et al., 2013; Karakasli et al., 2014; Donati et al., 2016; Eto et al., 2016; Piccioli et al., 2016; Scoccianti et al., 2016; Hashimoto et al., 2020; Sambri et al., 2020; Denes et al., 2022; Smolle et al., 2022). Five studies reported the occurrence of argyria when using the silver-coated megaprostheses (Hardes et al., 2010; Glehr et al., 2013; Karakasli et al., 2014; Denes et al., 2022; Smolle et al., 2022). The highest incidence of argyria was 23% (7/32) in Glehr et al.‘s study (Glehr et al., 2013), followed by 8.7% (4/46) in Smolle et al.‘s study (Smolle et al., 2022). Massè et al. conducted a study using external fixation with silver-coated stainless-steel pins to prevent pin tract infection. The researchers found that the postoperative blood silver levels increased to 3.12 ppb from the preoperative 0.2 ppb. This intervention was finally cancelled due to concerns about the significant increase in the concentration of silver in the blood (Massè et al., 2000). The other eight studies stated that none of the specific adverse events related to silver coatings was detected.

#### 3.3.2 Iodine coating

Six studies that reported the use of iodine coating were all conducted by the group of Shirai and Tsuchiya (Tsuchiya et al., 2012; Shirai et al., 2014a; Shirai et al., 2014b; Hayashi et al., 2015; Kabata et al., 2015; Shirai et al., 2019). The main concerns regarding the side effects of iodine coatings were the allergy and impairment of thyroid function. A total of 479 patients following different orthopaedic procedures (including trauma, spine surgery, arthroplasty, revision and tumor resection) received iodine-coated implants. Only one suspicious episode of allergy to iodine after arthroplasty revision was observed (Tsuchiya et al., 2012). No patients showed thyroid malfunction.

#### 3.3.3 Gentamicin coating

The major side effects of gentamicin coating involved allergy, nephrotoxicity and hepatotoxicity. The gentamicin-coated tibial nails were used in a total of 145 patients from the three studies (Fuchs et al., 2011; Moghaddam et al., 2016; Schmidmaier et al., 2017). The results showed no systemic or other general side effects. In the study by Fuchs et al., the researchers estimated the gentamicin serum levels and the values were below 0.3 μg/mL in all patients (Fuchs et al., 2011).

### 3.4 Quality assessment

Table 3 summarized the quality assessment of the cohort studies and the Newcastle-Ottawa rank represented high quality. The quality of the three case reports was considered high-quality (Supplementary Table S2).

**TABLE 3:** The Newcastle-Ottawa Scale for the included cohort studies.

Authors	Selection				Comparability	Outcome			Total
	Representativeness	Non-exposed cohort	Ascertainment of exposure	Absence of the outcome		Assessment of outcome	Adequate duration	Accuracy	
Smolle et al	*	-	*	*	-	*	*	*	6
Sambri et al	*	*	*	*	-	*	*	*	7
Shirai et al	*	-	*	*	-	*	*	*	6
Schmidmaier et al	*	-	*	*	-	*	*	*	6
Moghaddam et al	*	-	*	*	-	*	*	*	6
Eto et al	*	-	*	*	-	*	*	*	6
Piccioli et al	*	-	*	*	-	*	*	*	6
Donati et al	*	*	*	*	-	*	*	*	7
Scoccianti et al	*	-	*	*	-	*	*	*	6
Hayashi et al	*	*	*	*	-	*	*	*	7
Kabata et al	*	-	*	*	-	*	*	-	5
Shirai et al. (megaprostheses)	*	-	*	*	-	*	*	*	6
Shirai et al. (pins)	*	-	*	*	-	*	*	-	5
Glehr et al	*	-	*	*	-	*	*	*	6
Hussmann et al	*	-	*	*	-	*	*	*	6
Tsuchiya et al	*	-	*	*	-	*	*	*	6
Fuchs et al	*	-	*	*	-	*	*	*	6
Hardes et al	*	-	*	*	-	*	*	*	6
Hardes et al	*	-	*	*	-	*	*	*	6
Massè et al	*	*	*	*	-	*	*	-	5

## 4 Discussion

IRI is a disastrous complication faced by orthopaedic patients and surgeons due to the substantial morbidity and mortality, as well as heavy financial and psychological burdens. The formation of biofilm plays a critical role in the development of IRI, and various prophylactic methods against biofilm formation have been developed (Parvizi et al., 2017). Among them, antibacterial modification of implant surface has been proven as a powerful method for its promising antibacterial capability by numerous *in vitro*, *in vivo* and clinical studies (Wafa et al., 2015; Alt, 2017; Wang et al., 2021; Zhang et al., 2022; Zhao et al., 2022). However, there were only limited studies reporting the potential side effects of antibacterial coatings, and no systematic review has been published to summarize these concerns. To the best of our knowledge, the present systematic review is the first that identify the possible problems when using implants with antibacterial coatings. In order to demonstrate a comprehensive panorama of this topic, we also reviewed relevant case reports. In these studies, the main concerns of researchers regarding antibacterial coatings were a high concentration of antibacterial materials in blood, neurotoxicity, harm to organs/glands function, allergy and suppression on osteointegration. Based on available evidence, dermal discoloration was the most commonly-reported problem when using silver-coated implants. Iodine coatings might be associated with a possibility of anaphylactic adverse events, while the studies on gentamicin-coated nails did not report any side effects.

### 4.1 Silver

In our review, the main side effect of silver coatings was argyria, which was thought to be related to exposure to large amounts of silver (Lansdown, 2006). Silver has a long history of clinical use against infections (Alexander, 2009). It can cause harm to bacteria including membrane destruction, DNA condensation and so on (Aldabaldetrecu et al., 2018). However, an overdose of silver can damage host cells. Many clinical studies have shown the excellent antibacterial capability of silver-coated implants in orthopaedic procedures (Schmolders et al., 2017; Zajonz et al., 2017). Diez-Escudero et al. reviewed the published data on silver-coated arthroplasty components and found that silver coatings could reduce the risk of IRI, particularly in tumor patients with megaprostheses (Diez-Escudero and Hailer, 2021). However, silver-coated megaprostheses usually indicated a high silver content in the implant surface. This might explain why all reported argyria cases were those in whom the megaprostheses (MUTARS) were used. Nearly 1 G of silver was added to the surface of this megaprosthesis. The two highest incidences of argyria occurrence were 23% reported by Glehr et al. and 8.7% reported by Smolle et al. (Glehr et al., 2013; Smolle et al., 2022), respectively, and all patients in their studies received MUTARS reconstruction. Other silver-coated implants, such as PorAg megaC prostheses and Ag-HA prostheses (Eto et al., 2016; Scoccianti et al., 2016; Hashimoto et al., 2020; Sambri et al., 2020), contained a relatively lower amount of silver (0.33 g silver in PorAg megaC and 0.003 g silver in Ag-HA), and none of the cases with these implants had argyria. Fortunately, these reported cases of argyria were not associated with neurological deficits or systematic toxicity. Previous studies have identified that blood silver levels exceeding 300 ppb would lead to argyria, hepato- and nephrotoxicity (Noda et al., 2009; Ando et al., 2010). Among the five studies in our review that encountered argyria, two reported blood silver levels, ranging from 9.1 to 29.1 ppb (Denes et al., 2022; Smolle et al., 2022), and the occurrence of argyria was not related to the blood silver levels. Blood silver levels were also estimated in some other studies using PorAg MegaC prostheses or Ag-HA prostheses. Scoccianti et al. reported that the blood silver levels in patients receiving PorAg MegaC prostheses ranged from 0.82 to 20 ppb (Scoccianti et al., 2016). Eto et al. reported the clinical outcomes of Ag-HA prostheses and the blood silver levels ranged from 0 to 6 ppb (Eto et al., 2016). It seems that the blood silver levels may be lower in patients receiving implants with a relatively lower content of silver. Surgeons should monitor laboratory analyses and blood silver levels after the implantation of silver-coated prostheses. In addition, several *in vitro* and *in vivo* studies have shown that elevated silver ions might influence the activity of osteoblasts and thus inhibit osteointegration (Yonekura et al., 2011; Hauschild et al., 2015; Croes et al., 2018). We also noticed that the age of patients occurring argyria in our review was variable, ranging from 14 to 75 years, which might suggest that the occurrence of argyria was not associated with age. However, the elderly patients were at higher risk of organ dysfunction and thus should be treated carefully when they were about to receive antibacterial-coated implants. Silver has also been used in other clinical settings to prevent infections, such as wound dressings and bone cement (polymethylmethacrylate). These applications were also associated with several adverse events. Trop et al. reported that a burn patient represented argyria with liver dysfunction after using silver-coated dressings (Trop et al., 2006). Sudmann et al. reported a case of severe neurological paralysis after total hip arthroplasty with silver-impregnated bone cement (Sudmann et al., 1994). Although tthese severe side effects have not been reported when using silver-coated implants, they should also be concerned.

Apart from silver, other metals including zinc (Zn) and copper (Cu) are also investigated as antibacterial materials for surface modification. Zinc ions can inhibit bacteria by inactivating enzymes, destroying colonization plaques and so on (Osinaga et al., 2003). Copper ions can interact with bacterial membrane proteins and penetrate bacterial cells, inducing reactive oxygen species (Fan et al., 2021). Nevertheless, no clinical study has been published until now. Li et al. produced a coating with titania nanotubes incorporated with zinc and found that this coating could enhance bone formation and reduce bacterial adhesion in a rat model. However, if the zinc content was tripled in the coating, cytotoxicity would occur (Li et al., 2014). Several studies have demonstrated the promising antibacterial activity of copper in experiments (Ingle et al., 2014; Huang et al., 2018; Shimabukuro et al., 2020). However, even though copper is the essential nutrient required for normal body function, it is also toxic with a concentration-dependent feature. Prabhu et al. exposed rat ganglion cells to different concentrations of copper (10–100 μM) and found that significant cytotoxicity was observed and a higher concentration of copper revealed the maximum cytotoxicity (Prabhu et al., 2010). More studies should be conducted to find a balancing concentration of these metals between antibacterial capacity and cytotoxicity.

### 4.2 Iodine and gentamicin

Iodine has been widely used as an antibacterial substance in surgical hygiene, such as sterilizing operative areas and instruments (Lepelletier et al., 2020). It is also an indispensable component of the thyroid hormone. Therefore, after the implantation of an iodine-laden prosthesis in a patient, the thyroid hormone levels should be dynamically investigated. Our review reflected that none of the patients who received iodine-coated implants in the involved studies showed significant changes in thyroid function. As the possibility of iodine-induced allergy existed, Shirai et al. recommended preoperative patch tests to confirm the absence of allergy (Shirai et al., 2014a). Nevertheless, we noticed that the only case with suspected allergy also passed this test. Thus, careful assessment after implantation is also important. Previous *in vitro* studies also demonstrated concerns about toxicity of iodine, such as delaying healing (Taga et al., 2018). Schmidlin et al. found that higher concentrations of povidone-iodine could impair the differentiation of osteoblasts (Schmidlin et al., 2009). However, Shirai et al. tested the cytotoxicity of iodine-coated titanium using the fibroblasts and found that its toxicity was low and similar to that of normal uncoated titanium and stainless steel (Shirai et al., 2011). The results of non-clinical studies were still uncertain. Up to present, iodine-coated technologies or implants are not commercially available, and the published data of clinical studies were with a relatively small sample size. Studies with long-term follow-ups and larger sample sizes for iodine coatings are required.

Gentamicin belongs to the aminoglycoside group and its systematic use for infection prevention is gradually limited due to serious dose-dependent side effects, including nephrotoxicity and ototoxicity (Jiang et al., 2017). Other contraindications contain allergy to aminoglycoside, pregnancy, myasthenia gravis and so on. Nowadays, gentamicin is mainly added to bone cement to prevent infection (Cara et al., 2020). In the surface coatings, gentamicin is carried by PLLA to be released locally without high systematic doses. One of the three included studies reported the serum gentamicin levels and the value was below 0.3 μg/mL, which was recognized as the threshold of toxicity (Fuchs et al., 2011). In the study by Moghaddam et al., the authors also found that the serum gentamicin levels were lower than 0. 2 μg/mL. These results indicated that the gentamicin-coated surface generated by the dip process did not cause the accumulation of gentamicin. To date, gentamicin coatings are only available for tibial nails (Schmidmaier et al., 2006). This coating might be limited because of a relatively high gentamicin resistance of 2%–50% (Romanò et al., 2019), especially to *Staphylococcus aureus*, the main pathogen causing IRI. There are several *in vivo* and *in vitro* studies on PLLA-vancomycin, which is much more effective against Gram-positive bacteria. However, clinical applications of vancomycin coatings are lacking (Kankilic et al., 2011; Kankilic et al., 2014).

### 4.3 Limitations

There are several limitations of this systematic review. First, the methodology of systematic review may introduce bias due to the possibly unavoidable missing of relevant studies. However, we have registered this systematic review and followed the PRISMA guideline to complete a thorough search of four main databases. We tried our best to identify any reported side effects of antibacterial coatings. Second, due to the paucity of publications, we could not aggregate the data to perform a meta-analysis. Thus, the incidences of side effects, such as argyria, were given individually rather than as pooled results. The overall estimation of the occurrence of side effects was then compromised. Third, the study designs of the included studies were different and case reports were also involved in the present review, which jeopardised the level of evidence for the present systematic review.

## 5 Conclusion

The present data reporting the side effects of antibacterial coatings were inadequate. Based on the limited available evidence, the incidence of side effects was low. The most reported side effects of antibacterial coatings in clinical use were argyria with silver coatings, of which the incidence could even reach 23%. Coating-related argyria might be related to a high amount of silver (MUTARS). Other coatings such as iodine and gentamicin coatings are also concerned due to their potential toxicity, while no episode of relevant complications has been reported in clinical studies up to now. Even though few adverse events and no fatal complications of antibacterial coatings were observed, researchers should always be aware of the doses of antibacterial materials in blood as well as the potential side effects, such as systematic or local toxicity and allergy. Further clinical studies with longer durations, larger sample sizes and higher levels of evidence are appealed to demonstrate a more comprehensive summary of side effects in antibacterial coatings and to confirm their ability against IRI.

## Data Availability

The original contributions presented in the study are included in the article/Supplementary Material, further inquiries can be directed to the corresponding authors.
