# Supplementary material for: Potential side effects of antibacterial coatings in orthopaedic implants: A systematic review of clinical studies
Source: Front Bioeng Biotechnol. 2023 Feb 9;11:1111386. doi: 10.3389/fbioe.2023.1111386 (PMC9947536; doi:10.3389/fbioe.2023.1111386)
Supplement: Supplementary file 1 [file DataSheet1.DOCX]

Supplementary Material

Potential side effects of antibacterial coatings in orthopaedic implants: A systematic review of clinical studies

**Hua Li, Daofeng Wang, Wupeng Zhang, Gaoxiang Xu, Cheng Xu, Wanheng Liu, Jiantao Li***

*** Correspondence:** Jiantao Li: lijiantao618@163.com

# Supplementary Table

Supplement Table 1. Search strategy.

| Search strategy | |
| --- | --- |
| Database | Embase, PubMed, Web of Science and the Cochrane library |
| Combination | #1 AND #2 AND #3 AND #4 AND #5 |
| Deadline | Until October 31^st^ 2022 |
| #1 | bacteria OR bacterial OR bacterium OR pathogen OR biofilm OR infection OR infectious OR antimicrobial OR antibacterial |
| #2 | “surface modification” OR “modified surface” OR coating OR coated |
| #3 | implant OR prosthesis OR instrument |
| #4 | risk OR failure OR fail OR problem OR complication OR dilemma OR defect OR disadvantage OR deficiency OR discrepancy OR "negative effect" OR “negative result” OR “poor result” OR “side effect” OR “adverse event” OR toxicity OR toxic |
| #5 | orthopaedic |

Supplement Table 2. Details of the Joanna Briggs Institute Critical Appraisal Checklist for each case report.

| Authors | 1 | 2 | 3 | 4 | 5 | 6 | 7 | 8 |
| --- | --- | --- | --- | --- | --- | --- | --- | --- |
| Denes et al | N | Y | Y | Y | Y | Y | Y | U |
| Hashimoto et al | Y | Y | Y | Y | Y | Y | Y | Y |
| Karakasli et al | N | Y | Y | Y | Y | Y | Y | Y |

Y, yes; N, no; U, unclear.

1. Were patient’s demographic characteristics clearly described?

2. Was the patient’s history clearly described and presented as a timeline?

3. Was the current clinical condition of the patient on presentation clearly described?

4. Were diagnostic tests or assessment methods and the results clearly described?

5. Was the intervention(s) or treatment procedure(s) clearly described?

6. Was the post-intervention clinical condition clearly described?

7. Were adverse events (harms) or unanticipated events identified and described?

8. Does the case report provide takeaway lessons?
